# Factors Associated with Postoperative Recurrence in Inverted Sinonasal Papilloma: A Single-Center Cohort Study

**DOI:** 10.3390/jcm15197619

**Published:** 2026-10-01

**Authors:** Hironori Nakayoshi, Hitoshi Hirakawa, Taro Ikegami, Shogo Takeda, Tomoyo Higa, Masatomo Toyama, Hidetoshi Kinjyo, Shunsuke Kondo, Norimoto Kise, Shinya Agena, Naoki Wada, Fumi Kawakami, Mikio Suzuki

**Affiliations:** 1 Department of Otorhinolaryngology, Head and Neck Surgery, Graduate School of Medicine, University of the Ryukyus, Ginowan 901-2720, Okinawa, Japan; nakayoshi_fwl2@cs.u-ryukyu.ac.jp (H.N.); aoi23@cs.u-ryukyu.ac.jp (H.H.); ikegami@cs.u-ryukyu.ac.jp (T.I.); tttna1834@gmail.com (S.T.); h059834@cs.u-ryukyu.ac.jp (T.H.); puyoraer99110@gmail.com (M.T.); kouhouiinn@yahoo.co.jp (S.K.); norimoto7@gmail.com (N.K.); harugen3@yahoo.co.jp (S.A.); 2 Department of Pathology and Oncology, Graduate School of Medicine, University of the Ryukyus, 1076 Kiyuna, Ginowan 901-2720, Okinawa, Japan; wadan@cs.u-ryukyu.ac.jp; 3 Department of Pathology and Cell Biology, Graduate School of Medicine, University of the Ryukyus, 1076 Kiyuna, Ginowan 901-2720, Okinawa, Japan; fumi@cs.u-ryukyu.ac.jp

**Keywords:** inverted sinonasal papilloma, recurrence, frontal sinus, attachment-site hyperostosis, human papillomavirus, viral load

## Abstract

**Background/Objectives:** Inverted sinonasal papilloma (ISP) is a benign tumor with clinically important risks of recurrence and malignant transformation. We investigated factors associated with postoperative recurrence and the clinical significance of human papillomavirus (HPV) DNA detection. **Methods:** We retrospectively reviewed 104 surgically treated patients with histologically confirmed ISP (2006–2024). Clinical and radiological factors, HPV genotype and viral load, and p16, p53, and Ki-67 expression were evaluated. Recurrence analysis included 86 patients with at least 12 months of total clinical follow-up. **Results:** Seven patients (8.1%) developed recurrence. Median total clinical follow-up in the recurrence-analysis cohort was 46.5 months. Frontal sinus involvement and attachment-site hyperostosis were associated with recurrence in an exploratory two-variable Cox model, with wide confidence intervals. An additional model incorporating Krouse stage failed to converge. HPV DNA was detected in 21/104 tumors (20.2%): 11 low-risk and 10 high-risk HPV-positive tumors. Viral load was quantifiable in five tumors (0.3–65.0 E6 copies/ng genomic DNA); the remaining 16 were below the quantification limit of 0.25 copies/ng. No significant association between HPV status and recurrence was demonstrated. Two tumors showed p16 overexpression, including one high-risk HPV-positive tumor; no consistent HPV-related immunohistochemical pattern was identified. **Conclusions:** Frontal sinus involvement and attachment-site hyperostosis were associated with recurrence in this cohort. With only seven events, the findings are exploratory and require validation. A clinically relevant HPV association cannot be excluded, and HPV transcriptional activity cannot be inferred from these data.

## 1. Introduction

Inverted sinonasal papilloma (ISP) is a benign epithelial neoplasm arising from the Schneiderian mucosa of the nasal cavity and paranasal sinuses. Although histologically benign, ISP is characterized by locally aggressive growth, a tendency toward postoperative recurrence, and an established risk of malignant transformation to squamous cell carcinoma. The reported recurrence rate after surgical treatment is approximately 10–15% [1,2], while malignant transformation occurs in approximately 10% of cases [3,4,5]. Therefore, complete surgical resection and long-term postoperative surveillance remain essential in the management of ISP.

Advances in endoscopic sinus surgery (ESS) have substantially changed the surgical management of ISP, allowing direct visualization of the tumor and its attachment site while reducing the need for external approaches [6]. Nevertheless, postoperative recurrence remains an important clinical problem. Several factors have been proposed as predictors of recurrence, including advanced tumor stage, previous surgery, tumor location, and incomplete removal of the tumor attachment site [7]. In particular, involvement of anatomically challenging regions, such as the frontal sinus, may increase the risk of residual disease because complete visualization and surgical access to the attachment site can be difficult.

Identification and adequate treatment of the tumor attachment site are fundamental principles of ISP surgery. Focal or localized hyperostosis on computed tomography (CT) is frequently observed at the site of tumor attachment and can provide an important radiological landmark for surgical planning. Hyperostosis at the attachment site may also identify an area in which meticulous removal of the involved mucosa and treatment of the underlying bone are required. Thus, specific anatomical and radiological characteristics of the attachment site, rather than tumor extent alone, may have important implications for postoperative recurrence.

In addition to these surgical and anatomical factors, biological factors potentially associated with the development and recurrence of ISP have been investigated [5,6,8]. Human papillomavirus (HPV) has long been proposed as a possible etiological factor. Although HPV DNA has been detected in a subset of ISPs [9], its clinical significance, particularly with respect to recurrence, remains controversial [10,11,12]. Importantly, detection of HPV DNA by highly sensitive polymerase chain reaction (PCR) does not necessarily indicate biologically active infection [13]. Quantitative assessment of viral load together with evaluation of HPV-related cell-cycle proteins may therefore provide additional biological context beyond HPV DNA positivity alone.

The present study aimed to identify clinical, anatomical, and radiological factors associated with postoperative recurrence in a single-center cohort of patients with ISP, with particular attention to frontal sinus involvement and hyperostosis at the tumor attachment site. In addition, HPV infection was comprehensively evaluated by HPV genotyping, quantitative viral load analysis, and immunohistochemical assessment of p16, p53, and Ki-67 to determine whether HPV-related biological features were associated with recurrence.

## 2. Materials and Methods

### 2.1. Study Design and Patients

This retrospective study included 104 consecutive patients with histologically confirmed ISP who underwent surgical treatment at the University of the Ryukyus Hospital between 2006 and 2024. Patients with malignant transformation were excluded from the study cohort.

**Figure 1:**
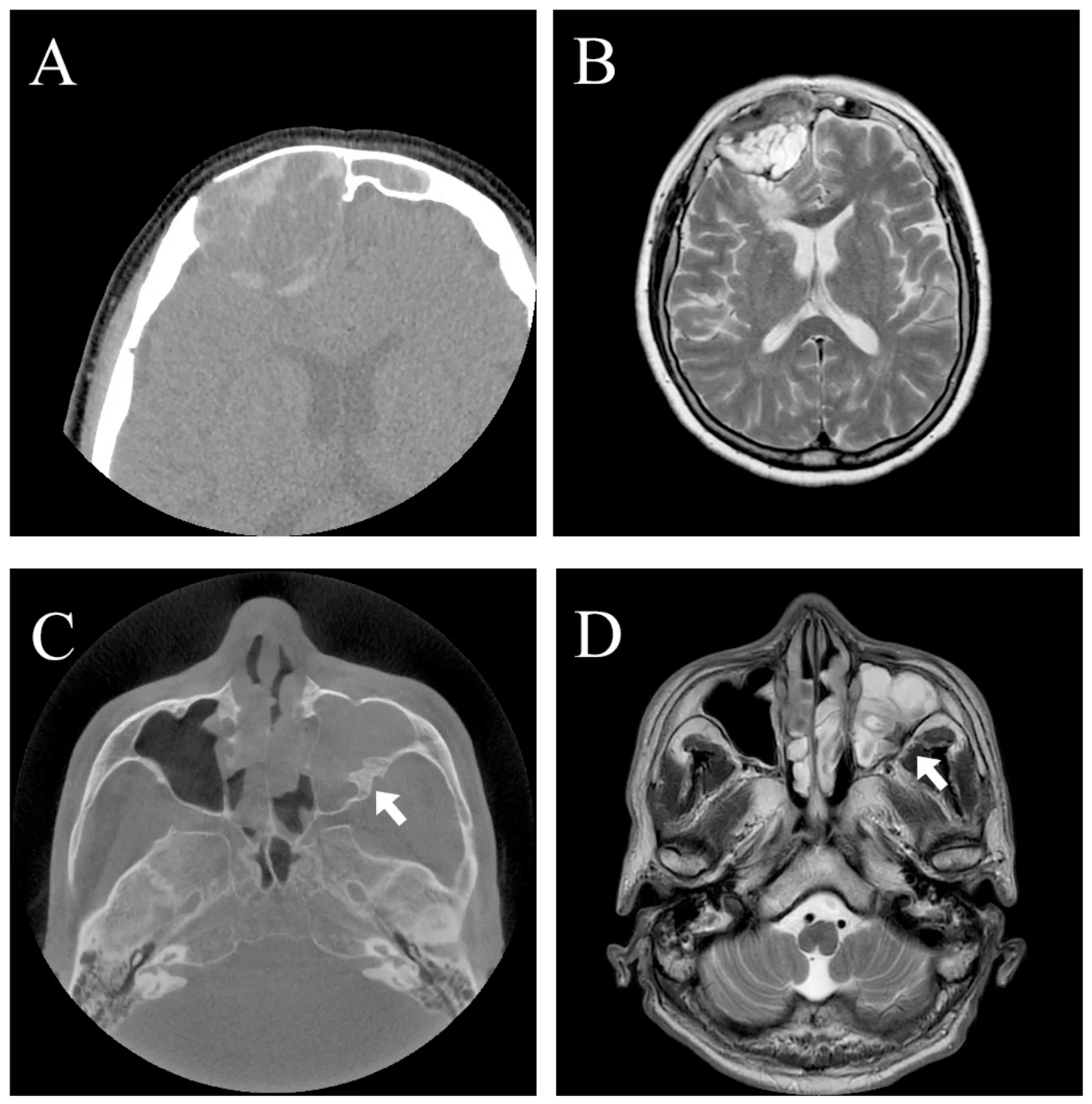
Representative imaging findings of frontal sinus involvement and hyperostosis at the tumor attachment site. (**A**) Axial non-contrast CT image showing an inverted papilloma occupying the frontal sinus, with bony defects of the anterior and posterior walls of the frontal sinus. (**B**) Axial T2-weighted MR image of the same patient as in (**A**), showing tumor involvement of the posterior wall of the frontal sinus. (**C**) Axial non-contrast CT image showing localized hyperostosis at the posterior wall of the left maxillary sinus (arrow), corresponding to the tumor attachment site. (**D**) Axial T2-weighted MR image of the same patient as in (**C**), showing a tumor occupying the left maxillary sinus. The arrow indicates the low-signal intensity area at the tumor attachment-site hyperostotic lesion shown on CT in (**C**).

Clinical variables included age, sex, smoking status, history of previous sinonasal surgery, serum squamous cell carcinoma antigen (SCCA) levels [14,15], Krouse stage [16], frontal sinus involvement, and hyperostosis at the tumor attachment site. All patients underwent preoperative computed tomography (CT), with magnetic resonance imaging (MRI) in selected cases. Preoperative CT and available MRI were retrospectively reviewed by three otorhinolaryngologists who were blinded to clinical and laboratory information, including recurrence outcomes and HPV results. Frontal sinus involvement and attachment-site hyperostosis were assessed on these images. Attachment-site hyperostosis was defined on CT as localized, eccentric thickening involving a limited portion of the bony wall of the affected nasal cavity or paranasal sinus (Figure 1).

Three patients with nasal exophytic papilloma were additionally examined as reference cases for comparison of HPV status, HPV viral load, and cell-cycle protein expression.

### 2.2. Surgical Treatment and Assessment of Recurrence

The surgical approach was selected according to tumor location, extent, and accessibility of the attachment site. ESS was the principal approach. Extended endoscopic procedures, including endoscopic medial maxillectomy, Draf type III frontal sinusotomy, transseptal access with crossing multiple incisions (TACMI) [17], and a direct approach to the anterior and lateral part of the maxillary sinus (DALMA) [18], were performed when required according to tumor location and extent. Whenever technically feasible and safe, the tumor attachment site was completely removed and the underlying hyperostotic bone was drilled or removed. Macroscopic complete resection was assessed intraoperatively in all cases. Intraoperative pathological examination was performed selectively when residual disease was suspected. Microscopic margin status was not systematically assessable across the cohort and was therefore not included as a standardized variable in the recurrence analysis.

After surgery, patients were followed by endoscopic examination, supplemented by CT and/or MRI when clinically indicated. Lesions suspected of recurrence were biopsied for histopathological confirmation. The index surgery was defined as the operation at our institution evaluated in this study, including operations performed for disease recurring after previous treatment. The event of interest was the first histologically confirmed recurrence after the index surgery.

Total postoperative clinical follow-up was defined as the interval from the index surgery to the last documented clinical assessment, including observation after treatment of recurrence. The requirement for at least 12 months of postoperative follow-up referred to total clinical follow-up, not to a minimum recurrence-free interval. For Kaplan–Meier and Cox regression analyses, time was measured from the index surgery to the first postoperative recurrence. Patients without recurrence were censored at their last assessment confirming the absence of recurrence. Observation after the first recurrence was not included in these time-to-first-recurrence analyses. No deaths were observed during documented follow-up.

### 2.3. HPV DNA Detection and Genotyping

HPV DNA detection and viral load quantification were performed as described previously [19]. Genomic DNA was extracted from fresh-frozen tumor specimens whenever available using the Gentra Puregene Tissue Kit (Qiagen, Gaithersburg, MD, USA). When fresh-frozen tissue was unavailable, DNA was extracted from formalin-fixed, paraffin-embedded (FFPE) specimens.

The presence and integrity of extracted DNA were confirmed by PCR amplification of the β-globin gene using the PC04 and GH20 primers. A negative control (water) and a positive control consisting of DNA from the HPV-16-positive CaSki cell line were included in each amplification series. HPV DNA was detected by nested PCR using the degenerate consensus primer sets GP5+/GP6+ and MY09/MY11 targeting the L1 region of the HPV genome. PCR products of the expected size were purified and directly sequenced using an ABI Prism 3130xl Genetic Analyzer (Applied Biosystems; Thermo Fisher Scientific, Waltham, MA, USA). The obtained sequences were compared with reference HPV L1 sequences in GenBank using the Basic Local Alignment Search Tool (BLAST). The detected genotypes were classified as low-risk or high-risk, in accordance with the IARC classification [20,21]. HPV-6 subtyping by cloning was not performed in this study.

### 2.4. Quantification of HPV Viral Load

Samples positive for HPV DNA by L1-targeted nested PCR were further analyzed by quantitative real-time PCR (qPCR) targeting E6 to determine HPV viral load [19,22,23]. HPV E6 DNA copies were quantified using a CFX96 Touch Real-Time PCR Detection System (Bio-Rad, Hercules, CA, USA). Viral load was expressed as E6 copies per ng of genomic DNA. The lower limit of quantification (LLOQ) was 0.25 E6 copies/ng genomic DNA. HPV DNA-positive samples below this threshold were reported as below the limit of quantification (BLQ), rather than assigned a viral load of zero or reclassified as HPV negative.

### 2.5. Immunohistochemical Analysis

Immunohistochemical staining for p16, p53, and Ki-67 was performed according to previously reported protocols [19]. Briefly, 4 µm thick FFPE sections were deparaffinized in xylene and rehydrated through a graded ethanol series. Immunohistochemical staining for p16 was performed using the CINtec p16 Histology Kit (MTM Laboratories, Roche Applied Science, Penzberg, Germany) according to the manufacturer’s protocol.

For p53 staining, antigen retrieval was performed using RE7119 Epitope Retrieval Solution (Leica Biosystems, Nussloch, Germany). For Ki-67 staining, sections were autoclaved at 120 °C for 20 min in Tris-EDTA buffer (pH 9.0) containing 0.05% Tween 20. Endogenous peroxidase activity was blocked by incubation with 0.3% hydrogen peroxide in methanol for 20 min at room temperature. Immunoreactivity for p53 and Ki-67 was detected using the SAB-PO Kit (Nichirei Bioscience, Tokyo, Japan). After blocking nonspecific binding with 10% normal goat serum, sections were incubated overnight at 4 °C with anti-p53 antibody (clone Bp53.11; Progen Biotech, Heidelberg, Germany; 1:500 dilution) or anti-Ki-67 antibody (clone MIB-1; DAKO, Santa Clara, CA, USA; 1:100 dilution). Sections were subsequently incubated with a biotinylated secondary antibody and peroxidase-conjugated streptavidin. Immunoreactivity was visualized using 3,3′-diaminobenzidine, followed by hematoxylin counterstaining.

### 2.6. Evaluation of Immunohistochemical Staining

p16 immunoreactivity was evaluated according to the percentage of positive tumor cells (score 0, 0%; score 1, 1–10%; score 2, 11–40%; score 3, 41–70%; and score 4, 71–100%) and staining intensity (score 0, absent; score 1, weak; score 2, moderate; and score 3, strong). For this study, p16 overexpression was operationally defined as moderate-to-strong nuclear and/or cytoplasmic staining in more than 70% of tumor cells (extent score 4 and intensity score 2 or 3). The criterion was adapted from the approach used in HPV-associated oropharyngeal squamous cell carcinoma [24,25] to distinguish extensive, at least moderately intense staining from weak or focal immunoreactivity. It was not treated as a validated surrogate for transcriptionally active HPV infection in benign ISP.

p53 immunoreactivity was evaluated according to both distribution and staining intensity. Distribution was classified as score 0, absent; score 1, sporadic; score 2, clustered; or score 3, diffuse. Staining intensity was classified as score 0, absent; score 1, weak; score 2, moderate; or score 3, strong.

Ki-67 expression was similarly evaluated according to distribution and intensity. Distribution was classified as score 0, absent; score 1, restricted to the basal-to-parabasal layers; score 2, extending to approximately the middle third of the epithelium; or score 3, extending to more than two-thirds of the epithelium. Staining intensity was classified as score 0, absent; score 1, weak; score 2, moderate; or score 3, strong.

Immunohistochemical staining was assessed independently by three otorhinolaryngologists who were blinded to clinical information. When the three ratings differed, the median score was adopted as the final score for the relevant scoring parameter.

### 2.7. Statistical Analysis

Categorical variables were compared using Pearson’s chi-square test or Fisher’s exact test, as specified in the tables. The age cut-off of 58 years was the median age in the full 104-patient cohort. The preoperative SCCA cut-off of 3.4 ng/mL was the median among available preoperative measurements in that cohort. These same cut-offs were applied to the 86-patient recurrence-analysis cohort, using <58 versus ≥58 years and <3.4 versus ≥3.4 ng/mL, respectively. These were cohort-derived thresholds for exploratory comparisons, not externally validated clinical cut-offs.

**Figure 2:**
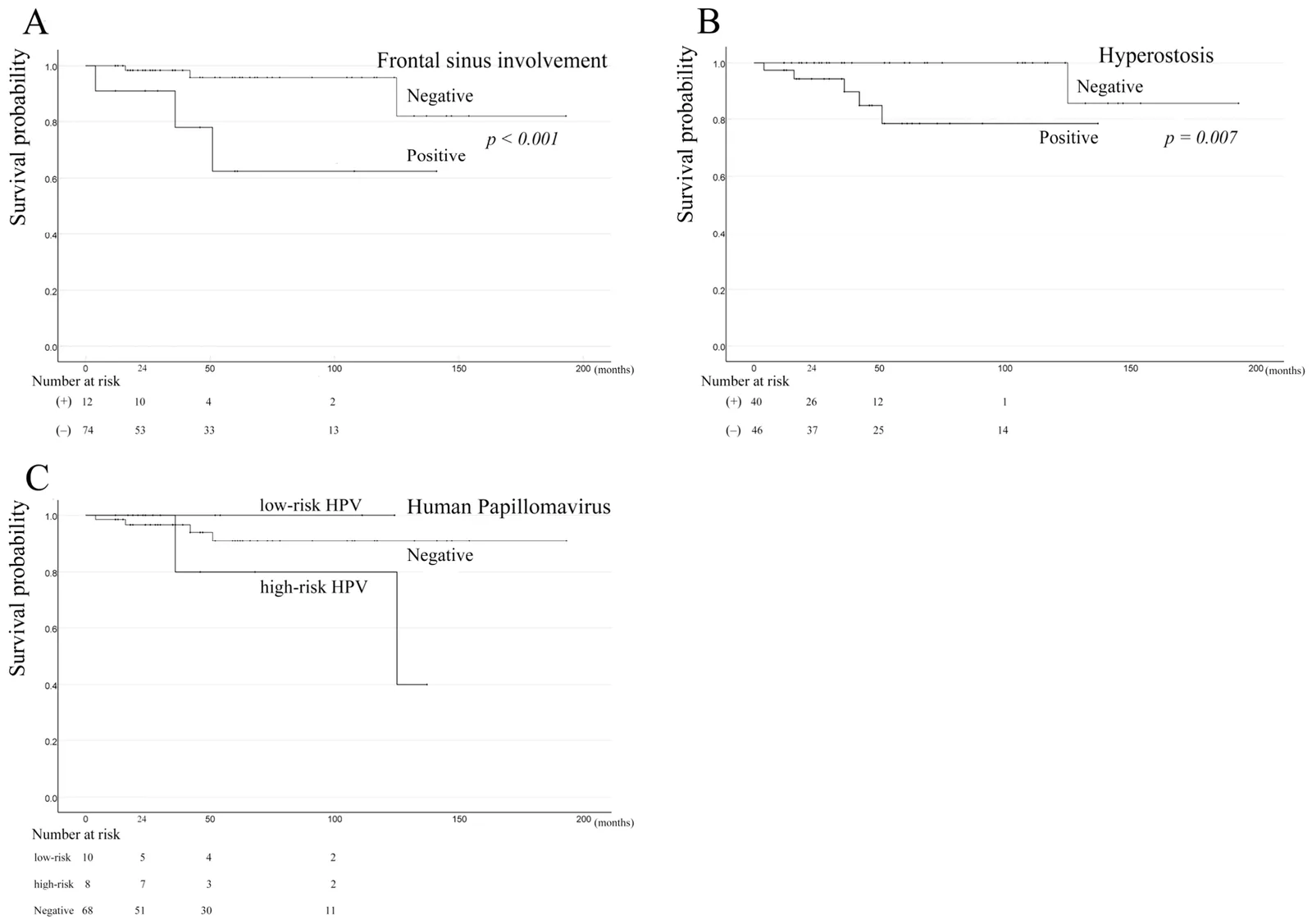
Kaplan–Meier curves for recurrence-free survival according to frontal sinus involvement, attachment-site hyperostosis, and human papillomavirus (HPV) status. (**A**) Patients without frontal sinus involvement had higher recurrence-free survival than those with involvement (log-rank test, *p* < 0.001). (**B**) Patients without attachment-site hyperostosis had higher recurrence-free survival than those with hyperostosis (log-rank test, *p* = 0.007). (**C**) Recurrence-free survival curves for patients with HPV-negative (*n* = 68), low-risk HPV-positive (*n* = 10), and high-risk HPV-positive (*n* = 8) tumors. Tick marks indicate censored observations. Numbers at risk are shown below each panel.

Recurrence-free survival was estimated using the Kaplan–Meier method, and group differences were evaluated using the log-rank test. An exploratory comparison of patients treated in 2006–2017 and 2018–2024 assessed possible treatment-era differences. HPV-related inference was based on two binary comparisons: HPV-negative versus any HPV-positive tumors, and HPV-negative/low-risk HPV-positive versus high-risk HPV-positive tumors. Figure 2C provides a descriptive display of the three HPV groups.

The reported exploratory Cox model included frontal sinus involvement and attachment-site hyperostosis to estimate their mutually adjusted associations with recurrence. The model was restricted to two covariates because only seven events occurred; this reduced model complexity but did not eliminate the risk of overfitting. Model convergence and the proportional-hazards assumption were checked. An additional conventional model incorporating binary Krouse stage (T1/T2 versus T3/T4) failed to achieve coefficient convergence, consistent with the sparse event distribution and possible monotone likelihood [26]. Its coefficient estimates and Wald *p* values were not used for inference. The reported two-variable estimates are therefore not adjusted for Krouse stage.

Hazard ratios (HRs) with 95% confidence intervals (CIs) are reported. All statistical tests were two-sided, and *p* < 0.05 was considered statistically significant. Statistical analyses were performed using IBM SPSS Statistics, version 28.0 (IBM Corp., Armonk, NY, USA).

### 2.8. Ethical Approval and Consent

The study protocol was approved by the Institutional Review Board of the University of the Ryukyus Hospital (approval nos. 156, R6.3–3, and R6.5–21) and was conducted in accordance with the Declaration of Helsinki. Written informed consent was obtained before collection of tissue samples. For the retrospective review of clinical records, study information was publicly disclosed on the website of the Department of Otorhinolaryngology, Head and Neck Surgery, University of the Ryukyus (https://ent-ryukyu.jp/, accessed on 1 August 2026), and patients were given the opportunity to opt out.

## 3. Results

### 3.1. Patient Characteristics and Surgical Treatment

A total of 104 patients with histologically confirmed ISP were included, comprising 78 men and 26 women. Baseline clinical characteristics according to HPV status are summarized in Table 1. Median total postoperative clinical follow-up was 35.0 months (interquartile range [IQR], 12.0–67.5; range, 0–214). Of these patients, 86 had at least 12 months of total clinical follow-up and were included in the recurrence analysis. In this cohort, median total clinical follow-up was 46.5 months (IQR, 23.75–71.5; range, 12–214). These follow-up periods included observation after treatment of recurrence.

Eighteen patients were excluded from the recurrence analysis because documented total clinical follow-up was shorter than 12 months. Most of these patients lived on other islands, and longer-term recurrence information could not be obtained despite attempts to contact them. Their available characteristics are compared with those of the 86 included patients in Supplementary Table S1. This descriptive comparison does not establish equivalence between the cohorts or exclude selection bias.

An endoscopic approach was used in 103 patients: 102 were treated entirely endoscopically, and one required an additional Caldwell–Luc approach because of intraoperative bleeding. One patient underwent repeat lateral rhinotomy for recurrent disease after a previous lateral rhinotomy because of the infratemporal tumor location and surrounding postoperative scarring. This repeat procedure was the index surgery for the present analysis, and the patient subsequently developed another recurrence, which was included among the seven postoperative recurrence events. Extended endoscopic procedures, including Draf type III frontal sinusotomy, endoscopic medial maxillectomy, DALMA, and TACMI, were used when required by tumor location and extent.

### 3.2. Postoperative Recurrence and Factors Associated with Recurrence

Among the 86 patients included in the recurrence analysis, seven (8.1%) developed histologically confirmed recurrence after surgery at our institution. The associations between clinical factors and postoperative recurrence are summarized in Table 2.

No significant association with recurrence was demonstrated for sex, age, smoking status, Krouse stage, preoperative SCCA level, history of previous sinonasal surgery, or HPV DNA status. Frontal sinus involvement and attachment-site hyperostosis were associated with shorter recurrence-free survival on Kaplan–Meier analysis (log-rank *p* < 0.001 and *p* = 0.007, respectively; Figure 2A,B). In the exploratory two-variable Cox model, the adjusted HR was 8.441 (95% CI, 1.805–39.467; *p* = 0.007) for frontal sinus involvement and 13.343 (95% CI, 1.276–139.515; *p* = 0.031) for attachment-site hyperostosis. Both estimates were imprecise. All seven recurrences occurred among the 59 patients with Krouse T3/T4 disease, whereas none occurred among the 27 patients with T1/T2 disease. The additional model incorporating Krouse stage did not converge.

In the 86-patient recurrence-analysis cohort, ESS was performed in 79 patients without recurrence and six with recurrence; the remaining patient, who underwent lateral rhinotomy, developed recurrence (two-sided Fisher’s exact test, *p* = 0.081). Only one patient underwent a non-ESS procedure, precluding a meaningful comparative assessment of surgical approaches. There were 43 patients in each surgical period: recurrence occurred in 3/43 patients treated in 2006–2017 (7.0%) and 4/43 treated in 2018–2024 (9.3%). No significant difference was demonstrated in observed recurrence proportions (two-sided Fisher’s exact test, *p* = 1.000) or recurrence-free survival (log-rank test, *p* = 0.168). These unadjusted comparisons do not exclude residual confounding by treatment era.

The observed time to first recurrence or censoring in the 86-patient cohort had a median of 40.5 months (IQR, 20.5–69.0; range, 4–193). Figure 2C displays the recurrence-free survival curves for the three HPV-status groups; the binary HPV comparisons are reported in Table 2.

### 3.3. HPV Prevalence and Genotype Distribution

HPV DNA was evaluated in all 104 ISPs. Fresh-frozen tumor specimens were available in 100 cases, whereas FFPE specimens were used for HPV analysis in the remaining four cases. HPV DNA was detected in 21 of 104 ISPs (20.2%): 11 tumors (10.6%) contained low-risk HPV and 10 (9.6%) contained high-risk HPV. No tumor harbored multiple HPV types. Among low-risk HPV-positive ISPs, HPV-6 was identified in six cases and HPV-11 in five; among high-risk HPV-positive ISPs, HPV-16 was detected in eight cases and HPV-33 in two (Table 3). Low-risk HPV DNA was detected in two of the three exophytic papillomas examined as reference cases.

Clinical characteristics according to HPV status are shown in Table 1. No significant differences were demonstrated in sex, age, smoking status, preoperative or postoperative SCCA levels, Krouse stage, frontal sinus involvement, or attachment-site hyperostosis among the three HPV groups. A history of previous sinonasal surgery was more frequent in the high-risk HPV-positive group (*p* = 0.012). Of the 21 HPV DNA-positive patients, case 11 (HPV-11) and cases 17 and 18 (HPV-16) were excluded from the recurrence analysis because total clinical follow-up was shorter than 12 months. Cases 2 and 9 had at least 12 months of total clinical follow-up and were included. The 86-patient cohort therefore comprised 10 low-risk HPV-positive, eight high-risk HPV-positive, and 68 HPV-negative patients. Two recurrences occurred among high-risk HPV-positive patients (cases 12 and 21), none among low-risk HPV-positive patients, and five among HPV-negative patients. No significant association between HPV status and recurrence was demonstrated.

### 3.4. HPV Viral Load

HPV viral load was assessed by qPCR in the 21 HPV DNA-positive ISPs (Table 3). Viral load was quantifiable in five tumors: one HPV-11-positive tumor (case 9, 0.3 E6 copies/ng genomic DNA) and four HPV-16-positive tumors (cases 12, 13, 15, and 16, with 1.5, 2.5, 65.0, and 22.7 copies/ng genomic DNA, respectively). The remaining 16 tumors were BLQ (<0.25 E6 copies/ng genomic DNA). The HPV-positive exophytic papilloma reference cases had viral loads of 4025 copies/ng for HPV-6 and 246,077 copies/ng for HPV-11. These reference cases were not included in either ISP cohort.

### 3.5. Immunohistochemical Findings

**Figure 3:**
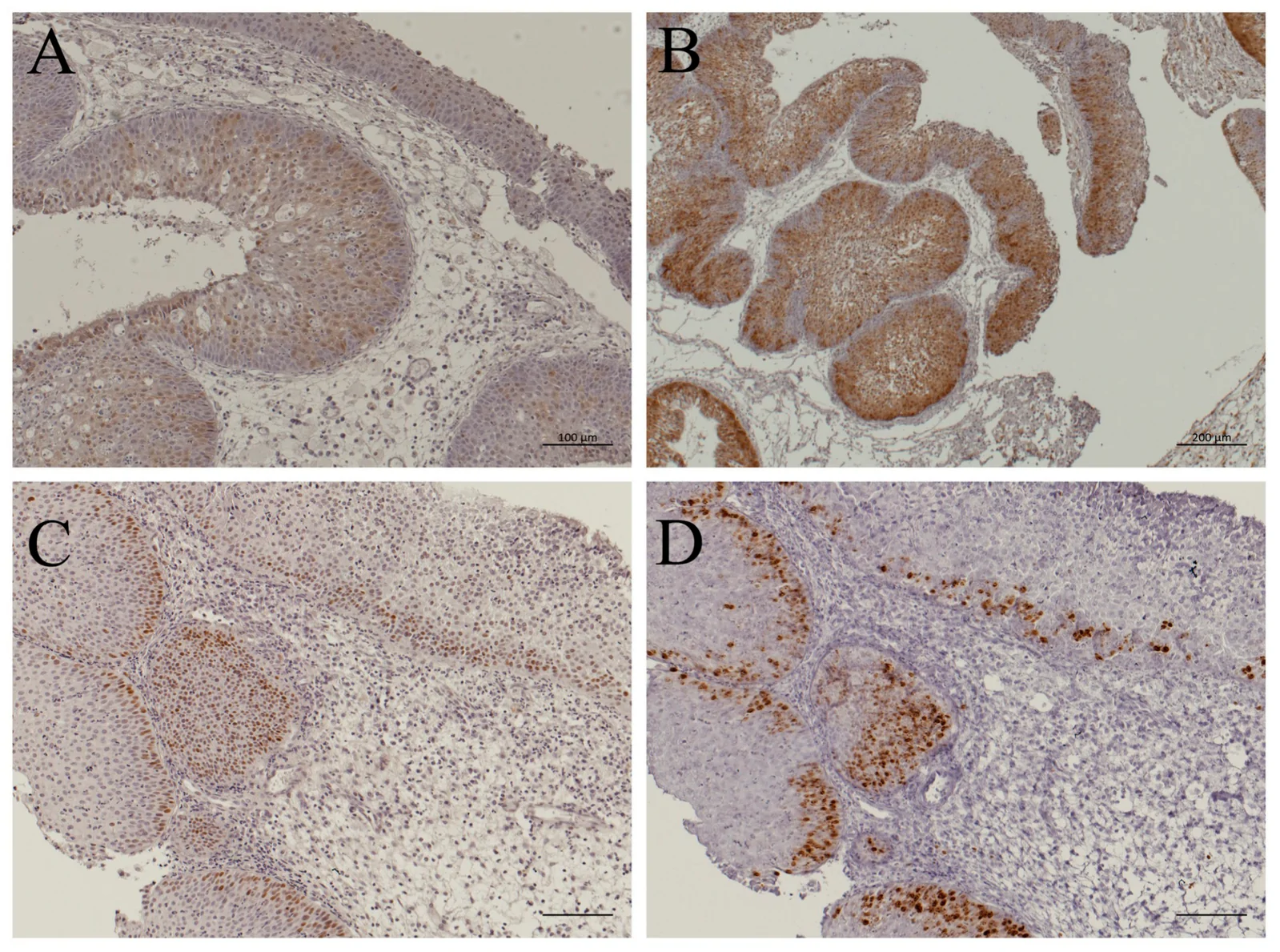
Representative immunohistochemical findings of p16, p53, and Ki-67 expression in inverted sinonasal papilloma. (**A**) p16 immunohistochemistry in an HPV-negative ISP, showing diffuse but weak p16 immunoreactivity. Scale bar = 100 μm. (**B**) p16 immunohistochemistry in a high-risk HPV-positive ISP, showing diffuse, moderate-intensity p16 immunoreactivity in >70% of the tumor epithelium (case 18 in Table 3). Scale bar = 200 μm. (**C**) p53 immunohistochemistry in an HPV-negative ISP, showing sporadic p53-positive cells from the basal to middle layers of the tumor epithelium. Scale bar = 100 μm. (**D**) Ki-67 immunohistochemistry in the same tumor as shown in (**C**), with strong Ki-67 immunoreactivity predominantly confined to the basal and parabasal layers. Scale bar = 100 μm.

p16 immunoreactivity was detected in 94 of 104 ISPs but was generally weak (Table 4; Figure 3A). Two tumors met the predefined criteria for p16 overexpression, showing moderate staining intensity in more than 70% of tumor cells. One was high-risk HPV-positive (case 18; Figure 3B), and the other was HPV-negative. Case 18 was excluded from the recurrence analysis because total clinical follow-up was shorter than 12 months.

p53 immunoreactivity was detected in seven cases and showed a sporadic distribution (Table 5; Figure 3C). One tumor with sporadic, strong p53 immunoreactivity subsequently recurred. Most tumors (97/104) showed no p53 staining.

Ki-67 immunoreactivity was detected in 101 of 104 ISPs and was predominantly localized to the basal and parabasal epithelial layers (Table 6; Figure 3D). Moderate-to-strong staining intensity was observed in 96 cases. No tumor demonstrated Ki-67 expression throughout the entire epithelium. Within the present sample size, no characteristic pattern of p16, p53, or Ki-67 expression distinguished high-risk HPV-positive from HPV-negative ISPs.

The three exophytic papilloma reference cases showed weak-to-moderate p16 immunoreactivity and Ki-67 expression confined to the basal/parabasal layers. p53 expression was absent in the two low-risk HPV-positive reference cases.

## 4. Discussion

In this retrospective single-center cohort of 104 patients with ISP, 86 had at least 12 months of total clinical follow-up and seven developed postoperative recurrence. Frontal sinus involvement and attachment-site hyperostosis were associated with recurrence in an exploratory two-variable Cox model. The estimates had wide confidence intervals and should not be interpreted as validated predictors. No significant association between HPV DNA detection, including high-risk HPV detection, and recurrence was demonstrated; however, the small number of HPV-positive tumors and events limits the ability to exclude a clinically relevant association.

The observed recurrence proportion was 8.1%, within or slightly below the range reported in previous studies [1,2]. Advances in endoscopic techniques facilitate identification and treatment of the attachment site, but local recurrence remains a concern. The minimum follow-up of 12 months does not ensure ascertainment of late recurrence, and observation after treatment of the first recurrence does not extend the time at risk for that first event. Therefore, the observed proportion should not be interpreted as a definitive long-term recurrence risk. Although no significant difference was demonstrated between the earlier and later surgical periods, only seven events occurred; the unadjusted period comparisons do not exclude changes in surgical practice or imaging as sources of confounding.

Frontal sinus involvement was associated with recurrence in this cohort. Surgical management of ISP involving the frontal sinus remains technically challenging because visualization and instrumentation of the attachment site may be limited by the frontal recess and sinus anatomy. In particular, tumors attached to the lateral or superior frontal sinus wall can be difficult to access completely through a conventional endoscopic approach [7]. Extended frontal sinus procedures, including Draf type III frontal sinusotomy, can improve access, although the attachment location and extent remain important considerations. The observed association supports careful preoperative assessment and sustained surveillance, but this study does not identify an optimal approach or prove that a particular technique reduces recurrence.

Attachment-site hyperostosis was also associated with recurrence. Focal hyperostosis on CT is a recognized radiological indicator of the origin or attachment of ISP [27]. Its potential relationship to the difficulty of achieving microscopic clearance is a proposed explanation, rather than a demonstrated mechanism. In this series, the attachment site and underlying hyperostotic bone were treated as extensively as was technically feasible and safe. Macroscopic complete resection was assessed in all cases, but microscopic margins and the extent of bone removal were not evaluated systematically. Consequently, the data cannot determine whether hyperostosis itself causes recurrence, whether it is a marker of difficult-to-treat attachment sites, or whether more extensive drilling prevents recurrence. Surgical implications must be considered alongside anatomical access and safety.

A second major aspect of this study was the comprehensive evaluation of HPV DNA. HPV DNA was detected in approximately one-fifth of tumors, including both low-risk and high-risk types, but no significant recurrence association was demonstrated. Previous studies have yielded conflicting results, with some reporting increased recurrence risk in HPV-positive tumors [12,28] and others finding no significant association [29]. Differences in detection methods, geographic populations, tissue preservation, and definitions of HPV positivity may contribute to this heterogeneity. The systematic assessment of all 104 tumors and quantitative testing of HPV-positive specimens provide additional context, but the present negative statistical findings do not establish the absence of an HPV effect.

At an LLOQ of 0.25 E6 copies/ng genomic DNA, viral load was quantifiable in five tumors (0.3–65.0 copies/ng), while the remaining 16 HPV DNA-positive tumors were BLQ. The earlier palatine tonsillar carcinoma series quantified HPV-16 and reported E6 copies per 50 ng of DNA [30]. After unit conversion, its range overlaps the HPV-16 values in the present cohort. Because the disease populations and assay conditions differ, those historical data are not used as a quantitative benchmark or as evidence of a uniform fold difference. Low-risk HPV-associated laryngeal papillomas examined using the same type-specific qPCR approach had higher reported viral loads [19], and the two exophytic reference lesions in the present study also had higher values. These are descriptive comparisons between distinct disease groups, not matched comparisons or thresholds for biologically active infection.

The distinction between HPV DNA detection and biologically active infection remains important. In established HPV-associated carcinomas, viral oncogene activity contributes to characteristic changes in cell-cycle regulation [31]. Low-copy HPV DNA in ISP could represent infection of a limited fraction of cells or incidental viral presence; these are hypotheses rather than conclusions from this study. Neither a low viral load nor a nonsignificant recurrence association establishes that HPV is transcriptionally inactive. Viral E6/E7 transcription was not assessed in the present cohort, and direct transcriptional assays would be needed to address this question.

Consistent with previous reports [13,32], p16 immunoreactivity was frequent but generally weak. Only two tumors met the study criteria for p16 overexpression, and one of these was high-risk HPV-positive. Ki-67 expression was predominantly confined to the basal and parabasal layers, while p53 expression was absent in most tumors; no consistent immunohistochemical pattern distinguished high-risk HPV-positive from HPV-negative ISPs. The p16 threshold was an operational research definition, not a validated surrogate for transcriptionally active HPV in benign ISP. In particular, the absence of p16 overexpression should not be interpreted as evidence against low-risk HPV involvement: the previous low-risk HPV-positive laryngeal papilloma cohort also showed low p16 expression and no clear association between viral load and clinical outcome [19]. The high-risk HPV-positive, p16-overexpressing case in the present study had less than 12 months of total clinical follow-up, so its recurrence outcome cannot be inferred from the 86-patient analysis.

A history of previous sinonasal surgery was more frequent among patients with high-risk HPV-positive tumors. Nevertheless, high-risk HPV positivity was not significantly associated with subsequent recurrence at our institution, and the clinical significance of the association with previous surgery is uncertain. Repeated intervention or persistent mucosal disease could influence the likelihood of HPV detection, but the timing of HPV acquisition was unknown and causality cannot be established. These data do not justify altering surgical extent solely on the basis of HPV DNA status.

The role of HPV in benign ISP recurrence must also be distinguished from its potential role in malignant transformation. HPV may have different biological significance in malignant lesions than in benign ISP [13,33]. Because cases with malignant transformation were excluded, the lack of a significant HPV-recurrence association should not be interpreted as evidence against HPV involvement in ISP-associated carcinogenesis. Studies including associated squamous cell carcinoma and assessing transcriptionally active HPV are needed to address that question.

Several limitations should be acknowledged. The retrospective, single-center design and long study period allow residual confounding by treatment era and unmeasured differences in surgical practice. Although images were reread with clinical and laboratory information masked, the study cannot fully separate anatomical associations from unmeasured microscopic residual disease or variation in bone treatment. Only seven recurrences occurred, resulting in imprecise estimates and potential overfitting. Limiting the model to two covariates did not remove those concerns. All seven events occurred in Krouse T3/T4 disease, and the additional stage-adjusted model failed to converge. Accordingly, the two-variable associations cannot be regarded as independent of Krouse stage.

Exclusion of 18 patients with short documented follow-up may have introduced selection bias, and a minimum of 12 months does not capture all late recurrences [34]. The descriptive comparison in Table S1 does not eliminate this bias. Only five HPV-positive tumors yielded quantifiable viral loads, limiting quantitative analyses, and no E6/E7 mRNA assay was performed. The p16 criterion was not validated for benign ISP, and the immunohistochemical observations cannot substitute for direct assessment of viral activity.

Despite these limitations, this study integrates clinical and radiological characteristics with HPV genotyping, quantitative viral-load assessment, and cell-cycle protein expression in the same cohort. Frontal sinus involvement and attachment-site hyperostosis are identifiable on preoperative imaging and merit evaluation in larger cohorts. The virological results add information beyond binary HPV DNA positivity, while underscoring the need to distinguish DNA detection from a demonstrated biological or prognostic role.

## 5. Conclusions

Frontal sinus involvement and attachment-site hyperostosis were associated with postoperative recurrence in this cohort. No significant association between HPV DNA detection and recurrence was demonstrated. Because only seven recurrences occurred and effect estimates were imprecise, the findings are exploratory and require validation in larger cohorts. A clinically relevant HPV association cannot be excluded, and the data do not establish viral transcriptional activity or a causal surgical mechanism.

## Figures and Tables

**Table 1:** Clinical characteristics of the 104 patients according to HPV status.

Variable	Total (*n* = 104)	High-Risk HPV Positive (*n* = 10)	Low-Risk HPV Positive (*n* = 11)	HPV Negative (*n* = 83)	*p* Value
**Sex**					0.776
Male	78	8	9	61	
Female	26	2	2	22	
Age at surgery, mean (years)	57.4	60.6	61.0	56.5	0.686
Total clinical follow-up, median (months)	35.0				
Range	0–214				
Interquartile range	12.0–67.5				
**Surgical procedure**					0.773
ESS only	102	10	11	81	
Other/combined approach	2	0	0	2	
**Previous sinonasal surgery**					0.012
Yes	31	7	2	22	
No	73	3	9	61	
**Preoperative SCCA**					0.999
No. tested	101	10	10	81	
>1.5 ng/mL	91	9	9	73	
≤1.5 ng/mL	10	1	1	8	
**Postoperative SCCA**					0.329
No. tested	97	9	11	77	
>1.5 ng/mL	20	1	4	15	
≤1.5 ng/mL	77	8	7	62	
**Smoking status**					0.837
Never	29	2	3	24	
Former/current smoker	75	8	8	59	
**Krouse stage**					0.056
T1/T2	34	2	7	25	
T3/T4	70	8	4	58	
**Frontal sinus involvement**					0.171 *
Negative	87	7	11	69	
Positive	17	3	0	14	
**Attachment-site hyperostosis**					0.629 *
Negative	52	5	7	40	
Positive	52	5	4	43	

Data are *n* unless indicated otherwise. ESS, endoscopic sinus surgery including extended endoscopic procedures; HPV, human papillomavirus; SCCA, squamous cell carcinoma antigen. *p* values compare the three HPV-status groups. Total clinical follow-up includes observation after treatment of recurrence. Follow-up summaries shown in the total column refer to all 104 patients. * For frontal sinus involvement and attachment-site hyperostosis, *p* values are from Pearson chi-square tests (2 degrees of freedom).

**Table 2:** Factors associated with postoperative recurrence in 86 patients with at least 12 months of total clinical follow-up.

Variable	No Recurrence (*n* = 79)	Recurrence (*n* = 7)	Contingency *p* Value	OR (95% CI)	Log-Rank *p* Value	Adjusted HR (95% CI)	Cox *p* Value
**Sex**			0.909		0.877		
Male	58	5					
Female	21	2					
**Age at surgery**			0.138		0.177		
<58 years	34	1					
≥58 years	45	6					
**Surgical procedure**			0.081 *		—		
ESS	79	6					
Other (lateral rhinotomy)	0	1					
**Surgical period**			1.000 *		0.168		
2006–2017	40	3					
2018–2024	39	4					
**Previous sinonasal surgery**			0.148		0.142		
Yes	24	4					
No	55	3					
**Preoperative SCCA**			0.645		0.790		
Number tested	77	7					
<3.4 ng/mL	37	4					
≥3.4 ng/mL	40	3					
**Smoking status**			0.309		0.398		
Never	26	1					
Former/current smoker	53	6					
**Krouse stage**			0.093		0.069	—	—
T1/T2	27	0					
T3/T4	52	7					
**Frontal sinus involvement**			0.006	11.83 (2.237–62.602)	<0.001	8.441 (1.805–39.467)	0.007
Negative	71	3					
Positive	8	4					
**Attachment-site hyperostosis**			0.046	7.941 (0.913–69.090)	0.007	13.343 (1.276–139.515)	0.031
Negative	45	1					
Positive	34	6					
**Any HPV DNA detection**			0.633		0.590		
HPV negative	63	5					
HPV positive	16	2					
**High-risk HPV**			0.126		0.178		
Negative/low-risk HPV	73	5					
High-risk HPV	6	2					

CI, confidence interval; HPV, human papillomavirus; HR, hazard ratio; OR, odds ratio. Counts refer to the 86-patient recurrence-analysis cohort. * Two-sided Fisher exact tests recalculated from the corrected counts. The surgical-procedure comparison is based on only one non-ESS patient; it does not establish equivalence of procedures. No procedure-specific log-rank or Cox estimate is supplied. Log-rank tests compare time to first postoperative recurrence; contingency tests compare observed recurrence proportions. Pearson chi-square *p* values are shown for sex, age, previous surgery, preoperative SCCA, and smoking; two-sided Fisher *p* values are shown for surgery, period, Krouse stage, frontal sinus involvement, hyperostosis, and HPV comparisons. Adjusted HRs are from the original two-variable model including frontal sinus involvement and attachment-site hyperostosis. The additional model with binary Krouse stage failed to converge and its Wald results. The two-variable estimates are exploratory and not adjusted for Krouse stage. Cut-offs were the full-cohort medians: age, 58 years; available preoperative SCCA, 3.4 ng/mL. Total clinical follow-up for all 86 patients: median 46.5 months (IQR, 23.75–71.5; range, 12–214), including surveillance after recurrence. Time to first recurrence or censoring: median 40.5 months (IQR, 20.5–69.0; range, 4–193).

**Table 3:** HPV genotype, viral load, immunohistochemical scores, and recurrence status in HPV-positive inverted sinonasal papillomas and exophytic papilloma reference cases.

Case	Sex	Age (Years)	HPV Type	Viral Load (Copies/ng Genomic DNA)	p16 Extent Score	p16 Intensity Score	p53 Distribution Score	p53 Intensity Score	Ki-67 Distribution Score	Ki-67 Intensity Score	Recurrence Status
1	M	77	HPV-6	BLQ	3	1	0	0	1	3	No
2	F	43	HPV-6	BLQ	4	1	0	0	1	3	No
3	M	78	HPV-6	BLQ	2	1	0	0	1	2	No
4	M	76	HPV-6	BLQ	2	1	0	0	1	3	No
5	M	50	HPV-6	BLQ	2	1	0	0	1	3	No
6	M	68	HPV-6	BLQ	3	1	0	0	1	3	No
7	M	65	HPV-11	BLQ	1	1	0	0	1	2	No
8	M	57	HPV-11	BLQ	2	1	1	1	2	3	No
9	F	40	HPV-11	0.3	2	1	0	0	1	3	No
10	M	62	HPV-11	BLQ	3	1	0	0	1	3	No
11	M	55	HPV-11	BLQ	2	1	0	0	1	3	No [a]
12	M	46	HPV-16	1.5	3	1	0	0	1	2	Yes
13	M	62	HPV-16	2.5	1	1	0	0	1	3	No
14	M	66	HPV-16	BLQ	3	1	1	2	1	3	No
15	F	54	HPV-16	65.0	2	1	0	0	1	2	No
16	M	70	HPV-16	22.7	4	1	0	0	1	3	No
17	M	53	HPV-16	BLQ	3	2	0	0	1	3	No [a]
18	M	36	HPV-16	BLQ	4	2	0	0	1	1	No [a]
19	M	80	HPV-16	BLQ	3	1	0	0	1	3	No
20	M	76	HPV-33	BLQ	2	1	0	0	1	2	No
21	F	63	HPV-33	BLQ	2	1	0	0	1	2	Yes
C-1	F	80	None	—	2	2	1	1	1	2	No
C-2	F	44	HPV-6	4025	1	1	0	0	1	2	No
C-3	M	83	HPV-11	246,077	2	2	0	0	1	2	No

[a] No recurrence was observed during the documented follow-up, but total clinical follow-up was shorter than 12 months; these patients (cases 11, 17, and 18) were excluded from the 86-patient recurrence analysis. Cases 2 and 9 had at least 12 months of total clinical follow-up and were included. The reference cases C-1 to C-3 are exophytic papillomas and are not part of either inverted-papilloma cohort. BLQ, below the lower limit of quantification (0.25 E6 copies/ng genomic DNA); F, female; M, male. Five HPV DNA-positive ISPs had quantifiable viral loads (one HPV-11 and four HPV-16); the remaining 16 were BLQ. BLQ does not indicate HPV negativity. A dash denotes no quantitative value for the HPV-negative reference case. p16 extent scores: 0, 0%; 1, 1–10%; 2, 11–40%; 3, 41–70%; 4, 71–100%. p53 distribution: 0, absent; 1, sporadic; 2, clustered; 3, diffuse. Ki-67 distribution: 0, absent; 1, basal/parabasal; 2, middle third; 3, more than two-thirds of the epithelium. Intensity for all markers: 0, absent; 1, weak; 2, moderate; 3, strong.

**Table 4:** p16 immunoreactivity in inverted sinonasal papilloma.

Percentage of p16-Positive Cells	Score 0: Absent	Score 1: Weak	Score 2: Moderate	Score 3: Strong
Score 0: 0%	10	0	0	0
Score 1: 1–10%	0	7 (1)	0	0
Score 2: 11–40%	0	33 (3)	0	0
Score 3: 41–70%	0	33 (3)	2 (1)	0
Score 4: 71–100%	0	17 (1)	2 (1)	0

Numbers in parentheses indicate tumors positive for high-risk HPV DNA. All counts refer to the full 104-patient cohort. Three otorhinolaryngologists scored staining independently while blinded to clinical information; the median score was used when ratings differed. Under the study definition, p16 overexpression requires an extent score of 4 and an intensity score of 2 or 3. Two tumors met this definition: one high-risk HPV-positive tumor (case 18 in Table 3) and one HPV-negative tumor. This is an operational research criterion, not a validated surrogate for active HPV infection in benign ISP.

**Table 5:** p53 immunoreactivity in inverted sinonasal papilloma.

Distribution of p53-Positive Cells	Score 0: Absent	Score 1: Weak	Score 2: Moderate	Score 3: Strong
Score 0: No staining	97 (9)	0	0	0
Score 1: Sporadic	0	5	1 (1)	1
Score 2: Clustered	0	0	0	0
Score 3: Diffuse	0	0	0	0

Numbers in parentheses indicate tumors positive for high-risk HPV DNA. All counts refer to the full 104-patient cohort.

**Table 6:** Ki-67 immunoreactivity in inverted sinonasal papilloma.

Distribution of Ki-67-Positive Cells	Score 0: Absent	Score 1: Weak	Score 2: Moderate	Score 3: Strong
Score 0: No staining	3	0	0	0
Score 1: Basal–parabasal	0	5 (1)	23 (4)	68 (5)
Score 2: Middle	0	0	1	4
Score 3: More than two thirds	0	0	0	0

Numbers in parentheses indicate tumors positive for high-risk HPV DNA. All counts refer to the full 104-patient cohort.

## Data Availability

The data that support the findings of this study are available from the corresponding author upon reasonable request. The data are not publicly available because of patient privacy and ethical restrictions.

## Supplementary Materials

The following supporting information can be downloaded at https://www.mdpi.com/article/10.3390/jcm15197619/s1: Table S1: Clinical characteristics of the full cohort and of patients included in or excluded from the recurrence analysis.
