# Supplementary material for: Factors Associated with Postoperative Recurrence in Inverted Sinonasal Papilloma: A Single-Center Cohort Study
Source: J Clin Med. 2026 Oct 1;15(19):7619. doi: 10.3390/jcm15197619 (PMC13666081; doi:10.3390/jcm15197619)
Supplement: Supplementary file 1 [file jcm-15-07619-s001.zip › jcm-4537600-supplementary.pdf]

Supplementary Materials

Factors Associated with Postoperative Recurrence in Inverted Sinonasal Papilloma: A Single-Center Cohort Study

Hironori Nakayoshi, Hitoshi Hirakawa, Taro Ikegami, Shogo Takeda, Tomoyo Higa, Masatomo Toyama, Hidetoshi Kinjyo, Shunsuke Kondo, Norimoto Kise, Shinya Agena, Naoki Wada, Fumi Kawakami and Mikio Suzuki

Table S1. Clinical characteristics of the full cohort and of patients included in or excluded from the recurrence analysis.

**Characteristic**	**Full cohort (n = 104)**	**Excluded: total clinical follow-up <12 months (n = 18)**	**Recurrence-analysis cohort (n = 86)**
**Surgical procedure**			
ESS	102 (98%)	17 (94%)	85 (99%)
Other	2 (2%)	1 (6%)	1 (1%)
**Previous sinonasal surgery**			
Yes	31 (30%)	3 (17%)	28 (33%)
No	73 (70%)	15 (83%)	58 (67%)
**Krouse stage**			
T1/T2	34 (33%)	7 (39%)	27 (31%)
T3/T4	70 (67%)	11 (61%)	59 (69%)
**Frontal sinus involvement**			
Negative	87 (84%)	13 (72%)	74 (86%)
Positive	17 (16%)	5 (28%)	12 (14%)
**Attachment-site hyperostosis**			
Negative	52 (50%)	6 (33%)	46 (53%)
Positive	52 (50%)	12 (67%)	40 (47%)
**HPV DNA status**			
HPV negative	83 (80%)	15 (83%)	68 (79%)
Any HPV positive	21 (20%)	3 (17%)	18 (21%)
Low-risk HPV positive	11 (11%)	1 (6%)	10 (12%)
High-risk HPV positive	10 (10%)	2 (11%)	8 (9%)

Values are n (%); percentages use the denominator in each column and are rounded to whole numbers. Rounding may prevent percentages from summing to 100%. ESS, endoscopic sinus surgery; HPV, human papillomavirus. Total clinical follow-up includes surveillance after treatment of recurrence.

The low-risk and high-risk HPV rows are subdivisions of the HPV-positive category, not additional patient groups. Low-risk types were HPV-6 and HPV-11; high-risk types were HPV-16 and HPV-33. The three HPV-positive excluded patients were case 11 (HPV-11) and cases 17 and 18 (HPV-16), as shown in Table 3.

The one non-ESS patient in the 86-patient cohort underwent lateral rhinotomy and subsequently developed recurrence after the index operation.

HPV categories are based on genotype detection, not on p16 staining or whether viral load was quantifiable. The three exophytic papilloma reference cases are not included in these ISP cohorts.
